# Folliculosebaceous cystic hamartoma of the vulva and groin: a rare case report

**DOI:** 10.3389/fmed.2026.1886298

**Published:** 2026-07-02

**Authors:** Xudong Chen, Chao Yuan, Xinghui Li

**Affiliations:** 1 Yancheng First Hospital, Affiliated Hospital of Nanjing University Medical School, Yancheng, China; 2 Hangzhou Adicon Clinical Laboratories Co., Ltd., Hangzhou, China

**Keywords:** case report, folliculosebaceous cystic hamartoma, groin, sebaceous hyperplasia, skin adnexal hamartoma, vulva

## Abstract

**Background:**

Folliculosebaceous cystic hamartoma (FSCH) is an uncommon benign adnexal hamartoma composed of follicular, sebaceous, and mesenchymal elements. It usually occurs on the head and neck, especially the central face, whereas genital and groin involvement is rare.

**Case presentation:**

A 49-year-old woman presented with a 20-year history of slowly progressive, multiple skin-colored papules and nodules arranged in a linear or beaded pattern on the right vulva and groin. She had no long-term medication exposure during the disease course. The lesions had no central pore, sinus, surface opening, expressible material, or protruding hairs. A local excisional biopsy performed on March 28, 2024 showed sebaceous hyperplasia-like changes with multiple mature sebaceous lobules. Because of subsequent enlargement, two larger nodules were locally excised on January 28, 2026. Histopathological examination demonstrated dilated infundibular cystic structures filled with keratinous and sebaceous debris, radially arranged mature sebaceous lobules connected to cystic spaces by sebaceous ducts, dysmorphic hair follicles, occasional apocrine structures, and proliferative collagenous stroma with adipose and vascular components. The final diagnosis was FSCH. No recurrence was observed at the 4-month clinical follow-up on May 28, 2026.

**Conclusions:**

This case highlights an uncommon genital and groin presentation of FSCH with multifocal, linear/beaded lesions and a long indolent course. It also illustrates how limited sampling may show only sebaceous hyperplasia-like features and delay recognition of the full diagnostic triad. Adequate excisional sampling and clinicopathological correlation are essential for diagnosis.

## 1 Introduction

Folliculosebaceous cystic hamartoma (FSCH) was first described by Kimura et al. in 1991 as a distinctive cutaneous malformation of the pilosebaceous unit (1). Histologically, FSCH is characterized by a cystically dilated follicular infundibular structure, radiating mature sebaceous lobules, and a mesenchymal stromal component composed of collagen, small blood vessels, and variably adipose tissue (1, 2).

Although FSCH was initially considered very rare, subsequent reports and case series have expanded its recognized clinicopathological spectrum. It most often presents as a solitary, asymptomatic, skin-colored papule or nodule on the head and neck, particularly the nose and paranasal region (2–4). Genital and groin presentations remain uncommon and may be clinically mistaken for more frequent vulvar or inguinal lesions, including condyloma, epidermoid cyst, sebaceous hyperplasia, fibroma, or other adnexal tumors (3, 5).

Herein, we report a 49-year-old woman with a 20-year history of multifocal FSCH involving the right vulva and groin. The case is notable for its rare location, linear/beaded configuration, long clinical course, and initial sebaceous hyperplasia-like biopsy finding caused by limited sampling.

This case report was prepared in accordance with the CARE guidelines for case reports (6).

## 2 Case presentation

### 2.1 Clinical history

A 49-year-old woman presented to the Department of Dermatology, Yancheng First People's Hospital, with a 20-year history of multiple skin-colored papules and nodules on the right vulva and groin. The lesions initially appeared without an obvious precipitating factor as rice-grain-sized papules and were asymptomatic. Over the subsequent two decades, they gradually increased in both size and number. The patient denied pain, pruritus, ulceration, discharge, infection, local trauma, prior surgery at the site, or similar lesions in family members.

During the 20-year disease course, the patient reported no long-term use of systemic corticosteroids, immunosuppressive agents, retinoids, oral contraceptives, or other medications temporally associated with lesion development. Her past medical history was otherwise unremarkable.

On March 28, 2024, a local excisional biopsy of a representative lesion was performed. The initial histopathological interpretation was sebaceous hyperplasia-like change. Because the lesions continued to enlarge, the patient returned and requested removal of the two largest nodules. Local excision of these nodules was performed on January 28, 2026.

### 2.2 Physical examination

**Figure 1:**
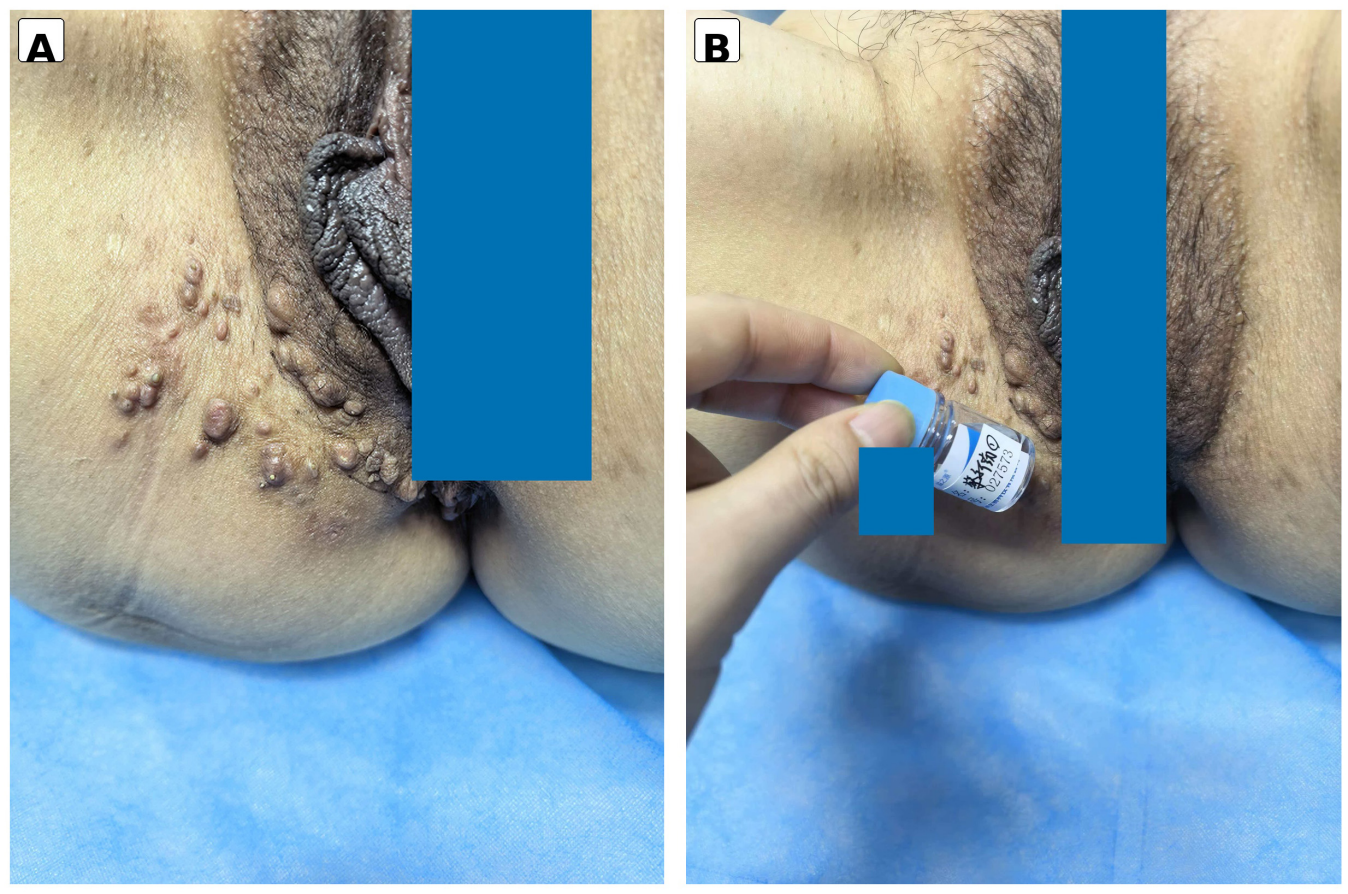
Clinical presentation. **(A)** Multiple flesh-colored papules and nodules distributed across the right vulvar and inguinal region in a linear/beaded pattern. **(B)** Close-up view showing clustered, dome-shaped papulonodules without a central pore, sinus, surface opening, expressible material, or protruding hair. Identifying information has been redacted.

**Figure 2:**
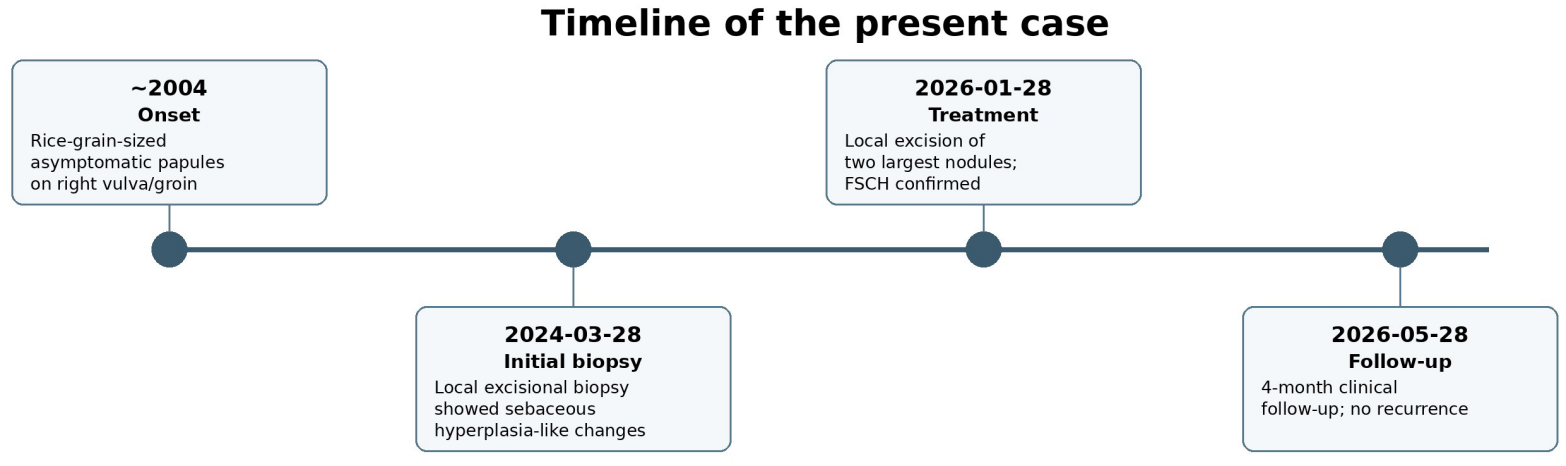
Timeline of the present case, including the approximate onset, initial local excisional biopsy, definitive local excision, and follow-up.

Dermatological examination showed multiple soft, skin-colored papules and nodules distributed in a linear or beaded pattern over the right vulvar and inguinal region (Figure 1). The lesions were non-tender and had no erosion, ulceration, or exudation. Careful inspection revealed no central pore, sinus tract, surface opening, expressible material, or protruding terminal/vellus hair. This absence of a central opening was clinically important in distinguishing FSCH from sebaceous trichofolliculoma and trichofolliculoma (Figure 2).

### 2.3 Histopathological findings

**Figure 3:**
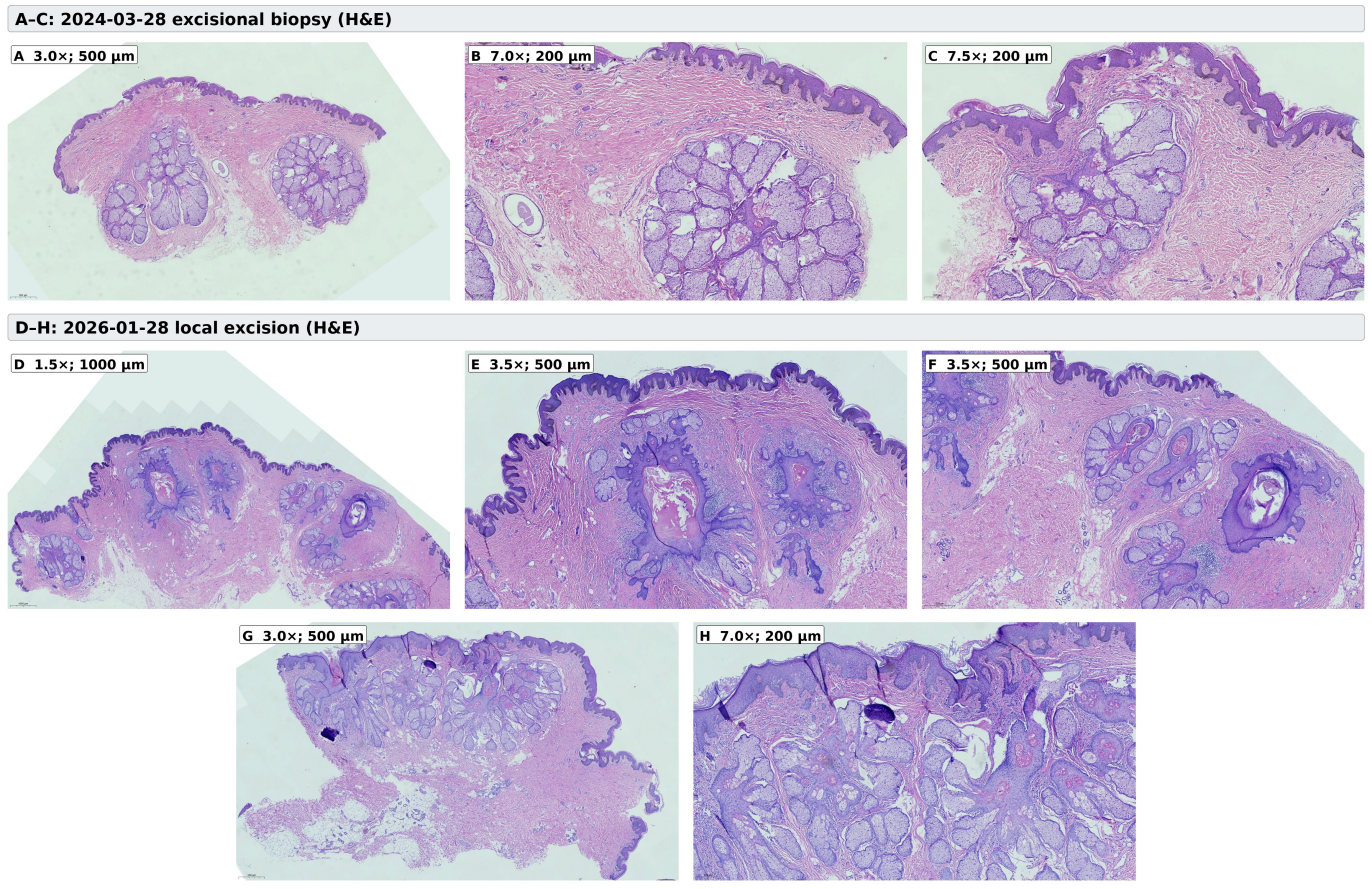
Histopathological findings of folliculosebaceous cystic hamartoma. **(A–C)** Local excisional biopsy performed on March 28, 2024. **(A)** Low-power view showing multiple mature sebaceous lobules within the dermis, surrounded by fibrous stroma (H&E, 3.0×; scale bar, 500 μm). **(B, C)** Higher-power views showing sebaceous lobules composed of peripheral basaloid germinative cells and central mature sebocytes, compatible with sebaceous hyperplasia-like changes; the limited specimen did not show the complete cystic or follicular diagnostic components of FSCH (H&E, 7.0× and 7.5×; scale bars, 200 μm). **(D–H)** Local excision performed on January 28, 2026. **(D)** Panoramic view showing multifocal dermal lesions with dilated infundibular cystic spaces, abundant radiating sebaceous lobules, and proliferative stromal components (H&E, 1.5×; scale bar, 1,000 μm). **(E)** Dilated infundibular cyst filled with keratinous and sebaceous debris, surrounded by radially arranged mature sebaceous lobules connected by sebaceous ducts (H&E, 3.5×; scale bar, 500 μm). **(F)** Cystic structure accompanied by dysmorphic hair follicles and focal inflammatory infiltrates (H&E, 3.5×; scale bar, 500 μm). **(G)** Low-power view of another section showing multifocal sebaceous and follicular components embedded in proliferative stroma (H&E, 3.0×; scale bar, 500 μm). **(H)** Higher-power view showing the classic triad of cystic follicular infundibulum, radiating mature sebaceous lobules, and surrounding collagenous stroma (H&E, 7.0×; scale bar, 200 μm).

The local excisional biopsy specimen obtained on March 28, 2024 showed multiple mature sebaceous lobules within the dermis, surrounded by fibrous stroma (Figures 3A–C). The available sections did not demonstrate the complete diagnostic combination of a cystically dilated infundibular structure, radiating sebaceous lobules, and characteristic mesenchymal stromal proliferation. Serial sections could not be performed retrospectively. Therefore, the 2024 diagnosis is best interpreted as a sampling-limited sebaceous hyperplasia-like component of the same clinical process, rather than proof that ordinary sebaceous hyperplasia progressed into FSCH.

The locally excised nodules obtained on January 28, 2026 showed the characteristic architecture of FSCH (Figures 3D–H). In the dermis, cystically dilated follicular infundibular structures were filled with keratinous and sebaceous debris. Numerous mature, markedly hyperplastic sebaceous lobules were radially arranged around the cystic spaces and communicated with them through sebaceous ducts. Dysmorphic hair follicles at various developmental stages were present, and occasional cystic apocrine structures were also observed. The surrounding stroma showed proliferative changes composed of collagen bundles, elastic fibers, adipose tissue, and vascular structures. No hair shafts were identified within the major cystic spaces. These findings established the diagnosis of FSCH.

Additional immunohistochemical marker studies, including CK17 and CK1/CK10, were considered because they can be helpful in selected difficult differential diagnoses. However, they could not be performed because no residual tissue was available for ancillary studies: the 2024 biopsy block contained no residual tissue for further sectioning, and the 2026 excision tissue had been entirely submitted for diagnostic H&E sections, with no remaining paraffin block available for immunohistochemistry. In the present case, the diagnosis was made on the basis of the classic H&E triad, namely cystically dilated follicular infundibular structures, radiating mature sebaceous lobules, and proliferative mesenchymal stroma.

### 2.4 Diagnosis

**Figure 4:**
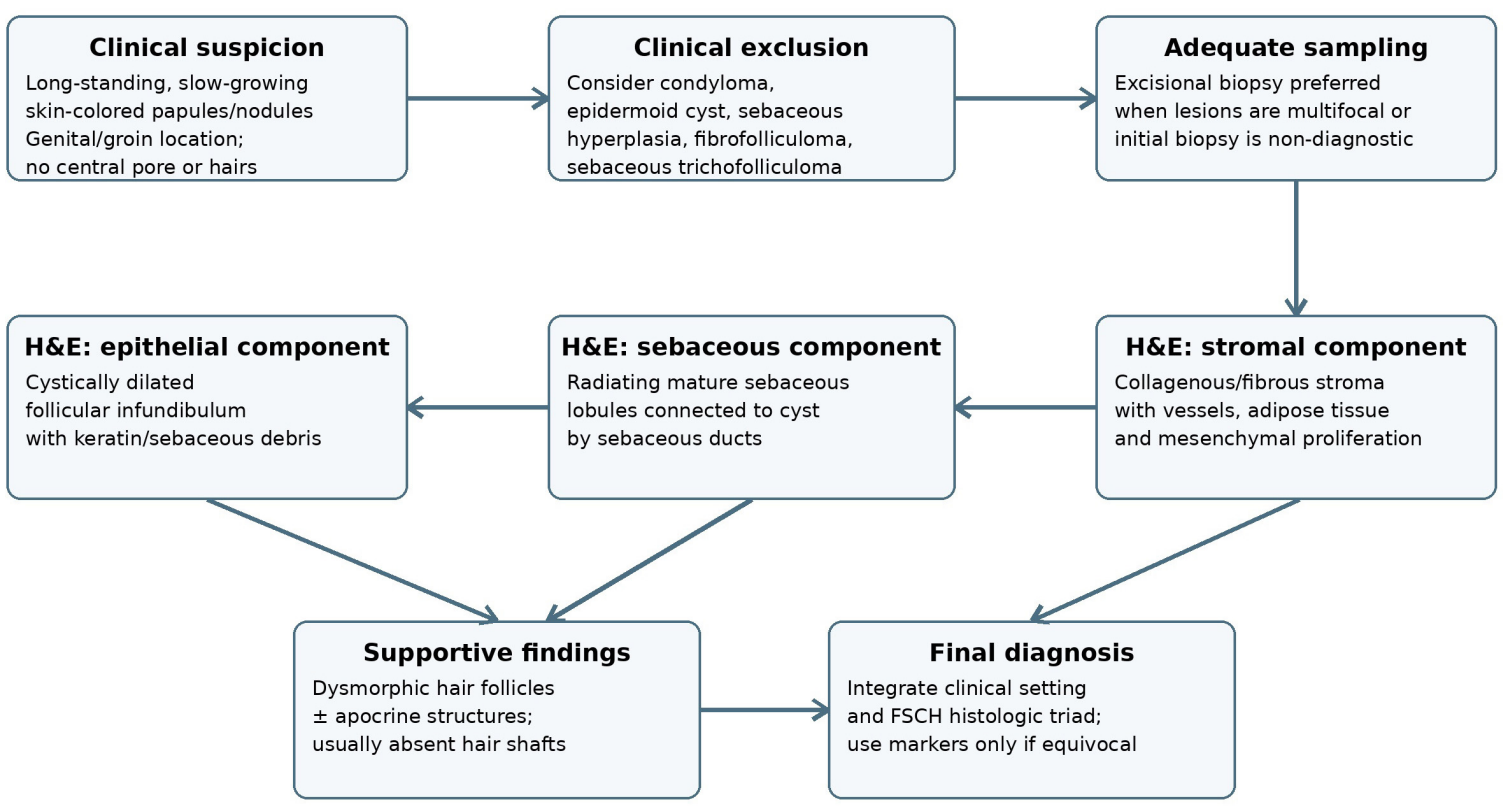
Diagnostic approach for suspected FSCH in genital or groin lesions. Adequate sampling and recognition of the epithelial, sebaceous, and stromal components are central to diagnosis.

Based on the clinical presentation and the histopathological triad of cystically dilated follicular infundibular structures, radiating mature sebaceous lobules, and proliferative mesenchymal stroma, a final diagnosis of FSCH involving the right vulva and groin was established (Figure 4).

### 2.5 Treatment and follow-up

The two largest nodules were completely removed by local excision under local anesthesia on January 28, 2026. The patient was informed that the lesion was benign and was advised to continue clinical follow-up because of the multifocal presentation. The last recorded follow-up was performed by clinical examination on May 28, 2026, 4 months after excision. No recurrence or new clinically significant lesion was observed at the excision sites or surrounding areas.

### 2.6 Patient perspective

The patient reported that she was satisfied with the surgical treatment and was reassured after learning that the lesion was benign. She agreed to continued follow-up.

## 3 Discussion

### 3.1 Epidemiology and significance of genital/groin involvement

FSCH is uncommon but is probably underrecognized because of its nonspecific clinical appearance. Kimura et al. first described five cases in 1991 (1). Before the large clinicopathological series by Ansai et al. was published in 2010, the number of reported cases in the English-language literature was fewer than 100; the abstract of that series specifically noted that only 70 cases had been reported after the original 1991 description (2). The 2010 series itself included 153 lesions, comprising 92 male and 61 female patients, giving a slight male predominance of approximately 1.5:1 (2). The age range was 15–88 years, with a mean age of approximately 54 years, and most lesions were solitary, small, and located on the face, particularly the nose (2). In the Introduction of a June 2023 case-based dermatopathology report, Urso and Yarygina summarized the subsequent literature and noted that more than 240 cases had been described over the preceding three decades, with more than 80% reported by Asian authors (4). Our targeted literature review was conducted in PubMed/MEDLINE and accessible full-text sources for publications from 1991 to 2026 and focused primarily on English-language literature. Additional cases may have appeared in non-English journals, including Japanese, Korean, and Chinese literature; therefore, case counts restricted to English-language publications may underestimate the true number of cases (3, 7).

Genital FSCH is particularly rare. The genital variant was first described by Bolognia and Longley (5). A concise review published in August 2022 reported that only six additional genital cases had been described after the original 1998 description, and the authors considered their case the eighth genital FSCH case known at that time (3). The present case contributes to this rare subset because it involved both the vulvar and groin regions, had multiple lesions, and followed an indolent 20-year course. A giant genital variant treated with carbon dioxide laser and acitretin has also been reported, further illustrating the clinical heterogeneity of genital FSCH (8).

### 3.2 Relationship between the 2024 biopsy and the final diagnosis

The initial 2024 biopsy showed only mature sebaceous lobules and was interpreted as sebaceous hyperplasia-like change. This finding should not be considered a separate disease process or definitive evidence of progression from conventional sebaceous hyperplasia to FSCH. Rather, it reflects the diagnostic limitations of small or partial sampling in a lesion whose defining features require recognition of the broader follicular, sebaceous, and stromal architecture. In the 2026 excision, the full lesion architecture was sampled and the diagnostic triad of FSCH was evident. This sequence emphasizes that excisional sampling should be considered when clinical lesions are longstanding, multifocal, anatomically unusual, or discordant with a limited biopsy diagnosis.

### 3.3 Differential diagnosis

The differential diagnosis can be approached first from the clinical presentation. In the genital region, multiple skin-colored papules and nodules may suggest condyloma acuminatum, epidermoid cysts, fibroepithelial polyps, sebaceous hyperplasia, or adnexal tumors. The absence of a verrucous surface, central pore, sinus, expressible keratinous material, and protruding hairs made condyloma, epidermoid cyst, sebaceous trichofolliculoma, and trichofolliculoma less likely clinically. Histopathological examination was required for definitive diagnosis. The histogenetic relationship between FSCH and trichofolliculoma remains debated. Some authors have proposed that FSCH may represent a late stage of trichofolliculoma, whereas other reports have documented the variable clinicopathological features of FSCH (9–11). Classic descriptions of trichofolliculoma, follicular neoplasms, and sebaceous trichofolliculoma emphasize a central follicular cavity, secondary follicles, and/or hair shafts, which help distinguish these entities from FSCH (12–15). Reports of giant FSCH and hereditary multiple fibrofolliculomas further underscore the need to distinguish FSCH from multiple or syndromic follicular tumors (16, 17). Standard dermatopathology references and subsequent histopathological, immunohistochemical, and clinicopathological studies support an integrated assessment of the follicular, sebaceous, and stromal components when diagnosing FSCH (18–21). Table 1 summarizes the major clinical and histopathological distinctions.

**Table 1:** Summary of differential diagnosis for vulvar/groin FSCH and related entities.

Entity	Clinical clues	Histopathological clues	Key distinction from the present case
FSCH	Usually solitary, slow-growing skin-colored papule/nodule; rare genital/groin involvement; may be clinically nonspecific.	Cystically dilated follicular infundibulum, radiating mature sebaceous lobules, and proliferative mesenchymal stroma with collagen, vessels, and fat.	The diagnostic triad (cystic infundibulum, radiating sebaceous lobules, and proliferative stroma) is characteristic; limited biopsies may show only sebaceous hyperplasia-like changes.
Epidermoid cyst	Subcutaneous or dermal nodule; may have punctum and express keratinous material.	Cyst lined by stratified squamous epithelium with granular layer and lamellated keratin; lacks radiating sebaceous lobules and stromal proliferation.	No central punctum or expressible material clinically; histology showed radiating sebaceous lobules and stromal proliferation.
Sebaceous hyperplasia	Small yellowish papules, usually on the face; often multiple but not typically cystic.	Enlarged mature sebaceous lobules opening into a central duct; lacks FSCH triad.	The 2024 biopsy showed sebaceous hyperplasia-like change, but the 2026 excision revealed the broader FSCH architecture.
Fibrofolliculoma	Firm, dome-shaped white/yellow papules; may be multiple, especially in syndromic settings.	Distorted follicle with radiating epithelial strands in fibrous or mucinous stroma; sebaceous differentiation may be focal.	The present case had abundant radiating mature sebaceous lobules and cystic infundibular spaces rather than the typical fibrofolliculoma pattern.
Sebaceous trichofolliculoma	Often has a central depression or opening with protruding vellus hairs.	Central follicular cavity with hair shafts and keratin debris; radiating sebaceous lobules; stromal proliferation is limited.	Present lesions lacked a central opening or protruding hairs, and major cystic spaces did not contain hair shafts.
Trichofolliculoma	Papule or nodule with central pore and one or more fine hairs, usually on the face.	Primary follicle opening to epidermis with multiple secondary follicles; hair shafts usually present.	Present case had the FSCH stromal component and lacked abundant hair shafts within the cystic spaces.
Condyloma acuminatum	Verrucous or cauliflower-like genital papules; may be multifocal.	Papillomatosis, acanthosis, koilocytosis, and HPV-related epithelial changes.	The lesions were dome-shaped rather than verrucous; histology did not show koilocytosis or HPV-related changes.

### 3.4 Linear/beaded configuration and Koebner phenomenon

The linear or beaded distribution of the lesions raised the question of whether Koebner phenomenon might be involved. In this patient, however, there was no history of local trauma, scratching, prior surgery, infection, or scar corresponding to the lesion distribution. Therefore, the available clinical information does not support a true Koebner phenomenon. The configuration may instead reflect local anatomic distribution along the vulvar-inguinal fold, mechanical friction, or clustering of lesions within adnexal-rich skin. Further cases would be needed to determine whether linear or segmental genital FSCH represents a reproducible clinical pattern.

### 3.5 Treatment, prognosis, and limitations

Complete surgical excision is generally considered the treatment of choice for FSCH and is usually curative (8). Alternative approaches, including carbon dioxide laser and oral retinoids, have been reported in selected cases, but efficacy remains variable and evidence is limited (8). In the present case, local excision of the two largest nodules achieved a favorable short-term outcome, with no recurrence at 4 months (Table 2).

**Table 2:** Summary of the major discussion points and clinical implications of the present case.

Discussion domain	Key points from the present case
Clinical significance	Uncommon vulvar/groin FSCH with multifocal linear or beaded lesions, a 20-year indolent course, and no central pore, sinus, surface opening, expressible material, or protruding hair.
Diagnostic implications	Adequate sampling is essential. The 2024 specimen showed only sebaceous hyperplasia-like changes, whereas the 2026 excision demonstrated the full H&E triad of cystically dilated follicular infundibular structures, radiating mature sebaceous lobules, and proliferative mesenchymal stroma.
Differential diagnosis	The main clinical and histological mimics include epidermoid cyst, sebaceous hyperplasia, fibrofolliculoma, sebaceous trichofolliculoma, trichofolliculoma, and condyloma acuminatum. Absence of a central opening or hair shafts and presence of stromal proliferation support FSCH.
Treatment and prognosis	Local excision of the two largest nodules was performed. No recurrence was observed at the last clinical follow-up 4 months after excision, but longer follow-up is recommended because the lesions were multifocal.
Limitations	The 2024 specimen could not be serially sectioned, immunohistochemical marker studies could not be performed because tissue was exhausted, and follow-up remains short.
Future directions	Additional genital/groin FSCH cases with long-term follow-up, serial sections, immunohistochemistry, and molecular characterization may clarify the pathogenesis and significance of multifocal or linear presentations.

This case has several limitations. First, the follow-up duration after excision was relatively short. The exact follow-up duration was 4 months, from January 28, 2026 to May 28, 2026, and longer surveillance is needed because the clinical presentation was multifocal. Second, serial sections of the 2024 specimen were not available, limiting retrospective reconstruction of the earliest histological features. Third, immunohistochemical marker studies could not be performed because no residual tissue was available for ancillary studies: the 2024 biopsy block contained no residual tissue for further sectioning, and the 2026 excision tissue had been entirely submitted for diagnostic H&E sections, with no remaining paraffin block available for immunohistochemistry. Future studies with longer follow-up, additional genital FSCH cases, and, where material permits, immunohistochemical or molecular characterization may help clarify the clinicopathological spectrum of this rare presentation.

## 4 Conclusion

We report a rare case of FSCH involving the vulva and groin, presenting as multiple linear/beaded papules and nodules over a 20-year course. The case emphasizes that limited biopsy may reveal only sebaceous hyperplasia-like changes and miss the complete FSCH triad. In longstanding, multifocal, or anatomically unusual genital lesions, adequate excisional sampling and careful clinicopathological correlation are essential for accurate diagnosis. The patient remained recurrence-free at 4 months after local excision.

## Data Availability

The original contributions presented in the study are included in the article/supplementary material, further inquiries can be directed to the corresponding author.
